# Interpretable Calibration Transfer and Drift Compensation for MOS Gas Sensors in Complex Gas Mixtures

**DOI:** 10.3390/s26144595

**Published:** 2026-07-20

**Authors:** Julian Schauer, Jannis Morsch, Dennis Arendes, Andreas Schütze, Christian Bur

**Affiliations:** Laboratory for Measurement Technology, Saarland University, 66123 Saarbruecken, Germany; j.morsch@lmt.uni-saarland.de (J.M.); d.arendes@lmt.uni-saarland.de (D.A.); schuetze@lmt.uni-saarland.de (A.S.); c.bur@lmt.uni-saarland.de (C.B.)

**Keywords:** interpretable machine learning, transfer learning, MOS gas sensors, calibration transfer, laboratory calibration, neural network representation

## Abstract

This study presents a novel approach for model-based calibration transfer and drift compensation for metal oxide semiconductor (MOS) gas sensors. The sensors are lab-calibrated and different calibration models for each of the eight volatiles contained in the calibration are trained to allow for an interpretable quantification of individual volatiles in complex mixtures. Calibration transfer and drift compensation are used to compensate for domain shifts that particularly affect the model accuracy. Here, several domain shifts are considered, e.g., sensor-to-sensor variation among different production batches (calibration transfer) or time-related changes in sensor response like poisoning and aging (drift compensation). Such domain shifts can lead to a substantial performance degradation and are critical for reliable field deployment. Since interpretable and robust machine learning algorithms based on feature extraction, feature selection, and regression (FESR) are not inherently capable of model-based calibration transfer and drift compensation, recalibration typically requires time-consuming and labor-intensive laboratory calibration procedures. To address this challenge, a novel approach represents the interpretable FESR machine learning models as a deep neural network (IDNNRep), enabling the application of transfer learning techniques from the field of deep neural networks (DNNs). This allows the reuse of knowledge gained in an initial calibration domain and facilitates model transfer using only a small amount of new calibration data, thereby reducing calibration effort and time. The proposed method is evaluated across multiple gases, including acetone and toluene, for four domain-shift scenarios and compared with FESR models retrained exclusively on data from the new domain and orthogonal signal correction (OSC). The results demonstrate that the proposed approach reduces the root mean square error (RMSE) compared to the initial model, achieving values of 18.0–28.0 ppb (normalized RMSE: 6.3–9.3%) for both gases with only 0.1 of the calibration data, resulting in a reduction of up to 93% compared to the initial calibration model and 89% compared to the OSC. Furthermore, due to the interpretable nature of the underlying FESR structure, the calibration transfer enables additional sensor- and gas-specific insights.

## 1. Introduction

According to the Global Burden of Diseases, Injuries, and Risk Factors Study (GBD) 2019 published in 2020, air pollution was responsible for approximately 6.7 million premature deaths worldwide in 2019 [1]. Reducing this substantial health burden requires a detailed understanding of both outdoor and indoor air quality (IAQ), particularly regarding toxic and harmful components. However, air represents a highly complex and dynamic mixture of gases and particulate matter, making comprehensive identification and quantification of all substances challenging [2,3]. For IAQ, accurately detecting volatile organic compounds (VOCs) which mainly affect the air quality is essential [4,5]. For indoor and especially outdoor monitoring applications, dense sensor networks are often needed to adequately cover the monitored space [6], which leads to the need for low-cost air monitoring systems. Most analytical measurement systems that enable highly accurate gas estimations [7] are costly, often require expert knowledge to operate, and represent spot measurements, which prevents widespread use, or are limited continuous online measurements [8].

Sensor systems are often based on metal oxide semiconductor (MOS) gas sensors as a cost-effective solution, enabling the development of compact real-time in situ sensor systems [9]. However, their inherent lack of selectivity limits their ability to quantify specific gases accurately. One approach is to use sensor arrays to increase the selectivity [10]. Alternatively, dynamic operation like temperature-cycled operation (TCO) has been proven to increase the selectivity and also improve sensitivity [11]. For IAQ, it was proven that the combination of a multipixel sensor and TCO allows quantification of single VOCs in complex mixtures with an uncertainty outperforming even standard analytics [12]. Based on complex lab calibration machine learning (ML)-based calibration models are trained for quantification of individual components in complex mixtures with up to 16 independent components [13]. The calibration model interprets complex sensor response patterns, especially in the transient response after temperature changes, and provides quantitative outputs as gas concentration [14]. The ML models are trained on calibration datasets recorded under controlled lab conditions using a gas mixing apparatus (GMA) capable of generating well-defined and complex gas mixtures [15]. However, responses from MOS sensors of the same type will typically exhibit slight differences. These changes in characteristics can be caused by minor changes in manufacturing [16], which slightly alter the actual hotplate temperature [17] or the behavior of the sensitive layers [18,19], especially when produced in a different production batch. Another challenge can occur when sensors are operated in the field, as the sensitive layers can drift over time [7,18,20]. Consequently, each sensor calibration model must be recalibrated to continue accurately estimating the target gas concentration, resulting in a time-consuming, high-effort calibration process.

Consequently, individual calibration procedures and dedicated calibration models are required for each sensor to ensure reliable gas estimation. This additional effort partially offsets the inherent cost advantage of low-cost MOS sensor technology.

Beyond robust model development based on feature extraction, feature selection, and regression (FESR) [21], previous studies have introduced calibration transfer and drift compensations approaches, including direct standardization and orthogonal signal correction, to reduce the calibration effort [22,23]. These methods compensate for systematic inter-sensor variations at an early stage of signal processing, i.e., manipulating the sensor raw data, enabling consistent predictions across different devices. While effective for small shifts in MOS sensor data, their performance decreases with more significant domain shifts [24]. Another drawback of direct standardization is that exactly the same test gas mixture is needed for the correction as used in the initial calibration, which is often not feasible. Significant domain shifts can be the transferring of a calibration model across different production batches (calibration transfer) or the accounting for changes in sensor responses over time (poisoning and sensor drift) due to irreversible alterations in the sensing layer (sub-sensor) of MOS gas sensors [25,26]. Reversible sensor drift is not considered in this study.

An alternative approach involves using deep neural networks (DNNs), which enable transfer learning (TL) by fine-tuning a pre-trained DNN calibration model with data from new sensors [26,27]. These model-based calibration techniques enable adaptation to a new domain after a significant domain shift with a reduced amount of calibration data from the new domain. Although DNN-based models can achieve high predictive accuracy and support model-based calibration transfer, they often operate as black boxes with limited interpretability [28] and require comparatively large datasets to train from scratch [29]. Additionally, the DNN exhibits reduced robustness to significant domain shifts, resulting in decreased accuracy without the collection of a novel calibration dataset [30]. Beside this, the introduced DNN requires high storage effort and computational complexity [31].

To address these limitations, a recent study proposed a novel framework that represents the inference procedure of an FESR model as dedicated DNN layers, allowing efficient implementation on edge hardware with a comparably small memory requirement [32]. This interpretable Deep Neural Network Representation (IDNNRep) approach preserves the interpretability and robustness of conventional FESR models while simultaneously improving computational efficiency and reducing inference time [33]. To reduce calibration time, the IDNNRep enables a model-based calibration transfer strategy based on TL without sacrificing transparency, by retraining specific layers of the IDNNRep to adapt to the novel domain. This method is explained in detail in the method section. In addition to introducing model-based calibration transfer, the novel approach allows interpretation of the changes observed during retraining. The interpretability of changes in the retrained model can facilitate deeper insights into sensor- and gas-specific behavior, providing valuable guidance for optimizing future calibration procedures and understanding the sensor’s sensitive layers.

In this context, the present work investigates the transferability of an initial FESR-based calibration model to sensors originating from a different production batch using a reduced set of unique gas mixtures (UGMs) for the transfer. Beyond inter-batch model transfer, this study also addresses recalibration of sensor-specific models to compensate for irreversible alterations in the sensitive sensor layer over time, leading to drift in sensor response and consequent degradation in quantification accuracy.

In this study, multiple initial FESR-based calibration models for different target gases are implemented as DNNs and subsequently transferred to a novel domain. This approach demonstrates the feasibility of model-based calibration transfer while preserving the interpretability of the underlying calibration models. The investigation focuses on two different gases out of a mixture of eight independent gas components and four different domain shifts. The proposed interpretable, model-based calibration transfer approach is evaluated across varying numbers of transfer samples, i.e., calibration points, and benchmarked against the performance of the initial calibration model, the OSC and an FESR model retrained exclusively on the corresponding transfer samples. In addition, the paper also investigates the interpretability of the calibration transfer and aims to gain novel insights into the various sub-sensors and the calibration process itself.

The paper is structured as follows: Section 2 describes the used dataset and methods, including the IDNNRep, the novel model-based calibration transfer, and the domain-shift scenarios used. Section 3 presents the results of the calibration transfer and interprets the changes obtained during that process. Section 4 discusses the results for the different gases, and Section 5 concludes the paper and gives an outlook on future work.

## 2. Materials and Methods

### 2.1. Dataset

**Figure 1:**
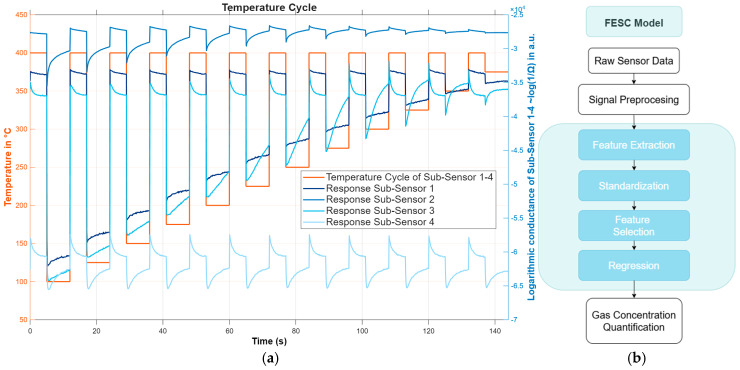
(**a**) Temperature cycle consisting of twelve temperature steps from 400 °C to a lower temperature in the range of 100 °C and 375 °C, combined with the responses of the four sub-sensors. (**b**) The scheme for the data processing of the raw data leading to the gas quantification.

For gas concentration quantifications based on MOS gas sensors and ML, the dataset used to calibrate the sensor model serves as the fundamental basis for achieving selectivity and accurate quantification. For this purpose, a custom-built GMA [16] was used to enable the generation of complex UGMs to generate calibration datasets for MOS sensors. The GMA allows arbitrary UGMs, each consisting of up to 16 independent gases over a wide concentration range from sub-ppb up to several tens of ppm without changing gas cylinders. The open-access dataset used in this study is published on Zenodo [34] and was acquired with this GMA and generated specifically to investigate the effects of batch variability and sensor drift over time. The dataset consists of two lab calibrations of seven MOS gas sensors, i.e., multipixel sensors SGP40, Sensirion AG, Staefa, Switzerland, with each sensor containing four gas-sensitive layers (sub-sensors). All sensors are operated in TCO (see Figure 1a) to enhance the sensitivity and selectivity of the sub-sensors. With temperature modulation, the sensor is never in a steady-state condition and transient responses are induced providing high-dimensional information [7]. Since, primarily, the shape of the sensor response changes in different mixtures, the use of form-describing feature extraction algorithms is favorable in the calibration model, see Figure 1b.

**Figure 2:**
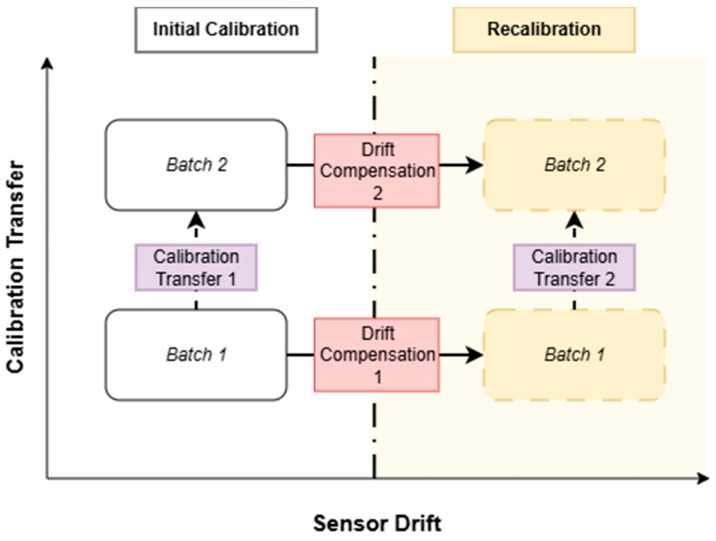
Overview of the four domain shifts, i.e., batch-to-batch, time shift batch 1, time shift batch 2 and batch-to-batch shift after one year of field operation, investigated in the dataset.

The dataset consists of two calibration runs with seven MOS gas sensors, each comprising 200 UGMs, where each UGM is represented by several temperature cycles, which results in a larger number of samples. Between these two calibrations, the sensors were operated in the field for one year leading to some sensor degradation and drift. Additionally, the seven sensors are from two production batches representing a second domain shift: three sensors are from batch 1 and four from batch 2, which also allows studying the effects of transferring calibration models between different batches. Since the data splitting is performed on the UGM level, the dataset is balanced in terms of the number of UGMs. The gases used in the two calibrations are listed in Table 1:

Since carbon monoxide and ethyl acetate are not included in the second calibration, this paper focuses on acetone and toluene as indicators for indoor air quality, which are contained in both calibration runs. Both gases are representative VOCs that are particularly relevant for indoor air quality (IAQ) monitoring. Acetone is commonly emitted from solvents and the human metabolism, while toluene is a widespread aromatic VOC originating from paints and coatings. Their distinct chemical properties and sensor response characteristics provide a representative basis for evaluating the proposed methodology. Results for the remaining gases included in the dataset are listed in Appendix A. The results are similar to those reported for acetone und toluene; the conclusions can therefore be generalized to other gases. Regarding the different sensor batches and the two calibrations, four relevant domain-shift scenarios are defined, as illustrated in Figure 2, excluding the combination of calibration transfer and sensor drift. Note that each scenario could, in principle, also be examined in the reverse direction, but this is out of the scope of this paper.

**Figure 3:**
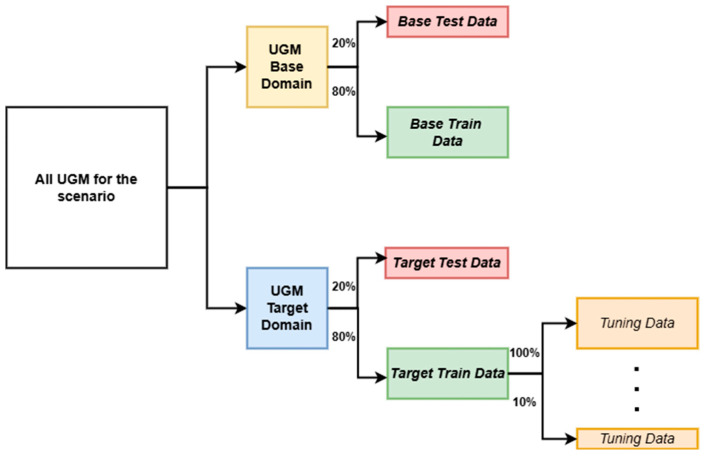
Scheme of the data evaluation splitting for the calibration transfer and the drift compenstation.

For each domain-shift scenario, the data are split into a base domain and a target domain. The base domain represents the origin of the data on which the calibration model was initially trained. The target domain captures the substantial changes the calibration model may encounter when transferred from a different batch to a sensor, as well as changes in the sensor response over time. In Figure 2, the base domain corresponds to the origin of the shift, i.e., the starting point of the arrow, while the target domain denotes its endpoint, i.e., the tip of the arrow. For each evaluation, the data splitting is performed as shown in Figure 3. Data splitting is based on UGMs, ensuring the model does not see the same UGMs in training and testing, i.e., has to interpret unknown gas mixtures in the test phase.

### 2.2. FESR Calibration Model

The underlying ML approach for the calibration model in this study is the FESR algorithm. These methods are available in the open-source automated Machine Learning Toolbox of the Lab for Measurement Technology at the Saarland University [35]. The FESR model enables the construction of a robust, interpretable regression model to quantify the target gas concentration. This method was used in several studies and achieved good results in estimating gas concentrations [13]. The goal is to learn the dependencies between the input data and the target gas concentration during training for all gases included in the calibration.

The feature extraction algorithm divides the data from the four sub-sensors of the SGP40 gas sensor into equidistant one-second segments. For each of these one-second segments, the mean and slope are calculated, resulting in 288 features per sub-sensor and a total of 1152 features. After calculating the form-describing features, all features are z-score standardized, i.e., resulting in a mean of zero and a standard deviation of 1 for each feature. In the following step, the most relevant extracted features are selected using Recursive Feature Elimination with Least Squares Regression (RFE-LSR) [36]. Since the FS and the regression are trainable parts, each gas is trained separately, which results in a single gas model. During training, the algorithm iteratively refines the feature set by evaluating regression coefficients and eliminating the least important feature. This allows the algorithm to find a suitable number of features by retaining the most significant ones. In the last step, Partial Least Squares Regression (PLSR) models the relationship between the selected features and the actual gas concentration by projecting them into a lower-dimensional latent space [37].

### 2.3. Interpretable Deep Neural Network Representation

**Figure 4:**
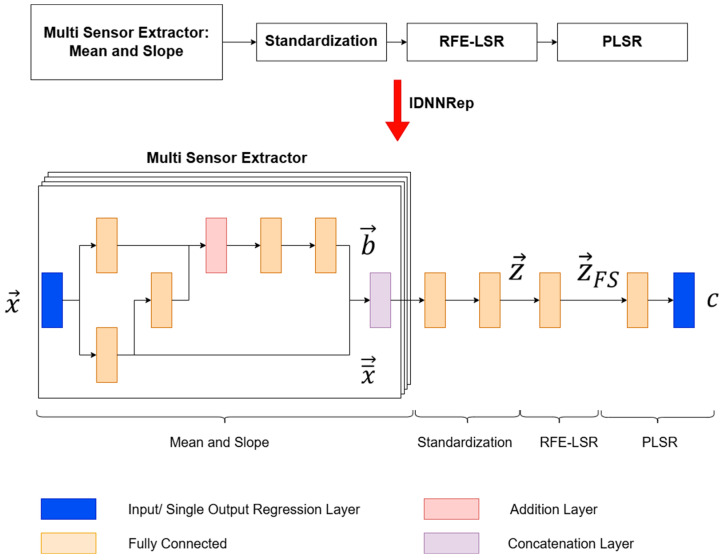
Interpretable IDNNRep of the FESR calibration model.

The IDNNRep of the inference of the FESR calibration model introduced in Section 2.2 is described in detail in the following sub-sections. For this purpose, the method breaks down the static inference into basic mathematical operations, such as multiplication, addition, and pooling. These basic operations, mostly based on matrix calculations, can then be implemented as neural network layers. In this section, each algorithm inference is first presented based on its fundamental computational formulation. Subsequently, its representation in terms of matrix–vector operations is provided, which can be directly mapped to the corresponding DNN layers (see Figure 4). This allows for understanding the architecture of the IDNNRep and the retraining process of the FESR calibration model.

#### 2.3.1. Feature Extraction

The mean ${\bar{x}}_{k}$ of each segment, as well as the combined means $\vec{\bar{x}}$ can be represented by the following matrix multiplication of $A_{r,c}$ with the input signal $x_{i}$ where k is the number of the segments, l the length of the segments and i the input signal length:

(1) $${\bar{x}}_{k}=\frac{1}{l}\sum_{j=v_{k}}^{v_{k+1}}x_{j},$$

(2) $$\vec{\bar{x}}=A_{r,c}\left[\begin{array}{l} x_{1}\\ x_{2}\\ \vdots \\ x_{i} \end{array}\right],\;where\;A_{r,c}=\left\{\begin{array}{l} \frac{1}{l},\;(r-1)l<c<rl\\ 0,\;\mathrm{otherwise} \end{array}\right.\;\in R^{\;k\times i}$$

Similarly, the calculation of the slope $b_{k}$ of each segment and the combined slopes ${\vec{b}}_{k}$ can be represented by the following equations, where $1_{l}\in R^{l}$ represents a vector of ones with length l:

(3) $$b_{k}=\frac{\sum_{j=v_{k}}^{v_{k+1}}\left(t_{j}-{\bar{t}}_{k}\right)\left(x_{j}-{\bar{x}}_{k}\right)}{\sum_{j=v_{k}}^{v_{k+1}}{\left(t_{j}-{\bar{t}}_{k}\right)}^{2}}$$

(4) $$\vec{b}=\frac{A_{r,c}\cdot \left[\left(\left[\begin{array}{l} x_{1}\\ x_{2}\\ \vdots \\ x_{i} \end{array}\right]-\left[\begin{array}{l} {\bar{x}}_{1}1_{l}\\ {\bar{x}}_{2}1_{l}\\ \vdots \\ {\bar{x}}_{k}1_{l} \end{array}\right]\right)⊙\left(\left[\begin{array}{l} t_{1}\\ t_{2}\\ \vdots \\ t_{i} \end{array}\right]-\left[\begin{array}{l} {\bar{t}}_{1}1_{l}\\ {\bar{t}}_{2}1_{l}\\ \vdots \\ {\bar{t}}_{k}1_{l} \end{array}\right]\right)\right]}{\left(\left[\begin{array}{l} t_{1}\\ t_{2}\\ \vdots \\ t_{i} \end{array}\right]-\left[\begin{array}{l} {\bar{t}}_{1}1_{l}\\ {\bar{t}}_{2}1_{l}\\ \vdots \\ {\bar{t}}_{k}1_{l} \end{array}\right]\right)⊙\left(\left[\begin{array}{l} t_{1}\\ t_{2}\\ \vdots \\ t_{i} \end{array}\right]-\left[\begin{array}{l} {\bar{t}}_{1}1_{l}\\ {\bar{t}}_{2}1_{l}\\ \vdots \\ {\bar{t}}_{k}1_{l} \end{array}\right]\right)},\;where\;\left[\begin{array}{l} x_{1}1_{l}\\ x_{2}1_{l}\\ \vdots \\ x_{k}1_{l} \end{array}\right]\in R^{k*l}$$

With the formulation as matrix multiplications, it is possible to represent the mean and slope calculation with neural network layers resulting in a layer graph as shown in Figure 4. The fractions presented in Equation (4) and thereafter represent the element-wise division of vectors, where the division operator is applied to each corresponding pair of entries.

#### 2.3.2. Standardization

The z-score standardization subtracts the mean of each feature and subsequently scales the result by the corresponding standard deviation. For a single feature, the standardized value z is computed as:

(5) $$z=\frac{feat-\mu }{\sigma },$$

where μ denotes the mean and σ the standard deviation of the feature, calculated during the training process. For the following calculations, the z-score standardization applied to each feature can be represented as a vector–vector subtraction and matrix–vector multiplication:

(6) $$\vec{z}=\left[\begin{array}{ccccc} \sigma_{1} & 0 & \cdots & 0 & 0\\ 0 & \sigma_{2} & \cdots & \cdots & 0\\ \vdots & \vdots & \vdots & \cdots & \vdots \\ 0 & 0 & 0 & \cdots & \sigma_{2k} \end{array}\right]\left(\left[\begin{array}{c} x_{1}\\ b_{1}\\ \vdots \\ x_{k}\\ b_{k} \end{array}\right]-\left[\begin{array}{c} \mu_{1}\\ \mu_{2}\\ \vdots \\ \mu_{2k-1}\\ \mu_{2k} \end{array}\right]\right),$$

#### 2.3.3. Feature Selection

The following feature selection reduces dimensionality and selects only the most significant features, using the RFE-LSR algorithm. For inference, the feature selection keeps only the n most significant features identified during training. Independent of the underlying algorithm, to select the most important features, each feature selection is implemented the same way and can be expressed with the following matrix–vectormultiplication, where $W_{FS}\in R^{n\;\times \;k}$ and each row of $W_{FS}$ contains exactly one non-zero element, with the value one:

(7) $${\vec{z}}_{FS}=W_{FS}\left[\begin{array}{l} z_{1}\\ \vdots \\ z_{2k} \end{array}\right]\;\in R^{n}\;$$

#### 2.3.4. Regression

The last part of the FESR algorithm is the PLSR. In the inference process, the PLSR’s linear transformation learned in training can be applied by multiplying the corresponding regression coefficients beta and adding the intercept, $\beta_{0}$, to quantify the target gas concentrations.

(8) $$c=\left[\begin{array}{ccc} \beta_{1} & \cdots & \beta_{n} \end{array}\right]{\vec{z}}_{FS}+\beta_{0}$$

#### 2.3.5. IDNNRep FESR Calibration Model

The final representation of the full FESR calibration model is a concatenation of the algorithms presented in detail above. The most commonly used layer is the fully connected layer, which implements vector–matrix multiplication using the weight matrix W and a subsequent bias addition, which can be initialized with the corresponding values. Besides this DNN layer type, one addition layer and one concatenation layer are used. Figure 4 represents the IDNNRep transforming the time series of all four sensor layers to the output concentration c of the target gas; a more detailed description can be found in [32]. Figure 4 also demonstrates that interpretability is preserved in the IDNNRep. The output of each layer can still be traced back to an interpretable value that explains sensor behavior and provides information about each sub-sensor’s input to the final concentration output.

### 2.4. Data Evaluation for the Calibration Transfer and Drift Compensation

**Figure 5:**
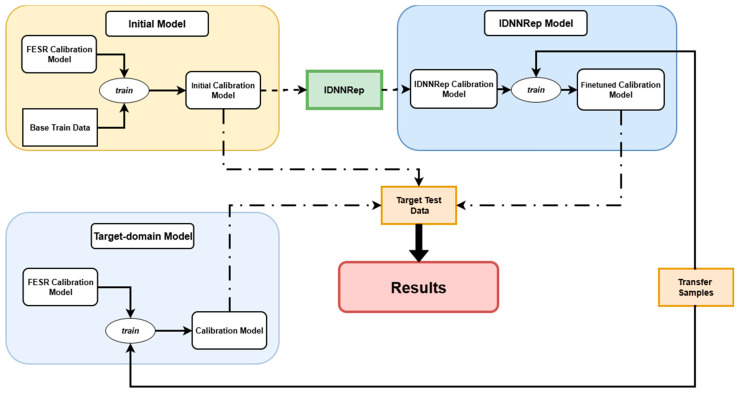
Scheme for evaluating the different models for calibration transfer.

The data evaluation of the calibration transfer and drift compensation is described in the following section, including the four significant domain shifts: two in the sensor batch and two in the time domain. To investigate the novel approach, an initial calibration model in the base domain is created, referred to as the initial model, as seen in Figure 5. This model is transformed into a neural network using IDNNRep and subsequently retrained on a split of the target-domain training data, as seen in Figure 3. For the retraining process, the feature extraction and standardization are frozen, and only the feature selection and the PLSR layers are retrained. This results in the retraining of two fully connected layers comprising approx. 288k learnable parameters, resulting in a comparatively low computational cost for model adaptation compared to the approach shown in [31]. The retraining process starts with a learning rate of $10^{-1}$ and drops the learning rate every 10 epochs with a learning rate drop factor of 0.9. As a result of the retraining process, the feature selection matrix $W_{FS}$, which originally consisted of binary values (0 and 1), changes during the retraining into a continuous weighting matrix with arbitrary real-valued coefficients while keeping the dimension $W_{FS}\in R^{n\;\times \;k}$. This enables the calibration model to adjust the contribution of previously selected features and to reintroduce features that were effectively excluded by the original feature selection step. The transformation of the binary feature selection weights into continuous feature weights is enabled by gradient-based optimization using standard backpropagation. During retraining, the original binary feature selection matrix $W_{FS}$ is fine-tuned to continuous values, allowing the contribution of each feature to be adjusted according to the target domain. Compared with a fixed binary feature selection, this increases the flexibility of the model by enlarging the optimization space, which can improve the adaptation performance while preserving the interpretable structure of the original FESR algorithm. In addition to the retraining of the feature selection layer, retraining the PLSR layer allows the regression coefficients to be adapted to the target domain, thereby further improving the model’s ability to account for domain-specific characteristics and enhancing overall transfer performance. During retraining, the PLSR layer no longer strictly preserves the variance-explaining properties of the original PLSR model, as its parameters are fine-tuned using backpropagation. Instead, the layer can be interpreted as a learned linear transformation that adapts the newly weighted features to the target-domain concentrations. This approach is compared with the standard approach of building a model from scratch, using the same FESR model architecture and the same tuning samples used for retraining the IDNNRep calibration model. This model is called the target-domain model. The train UGMs are split into 0.1 (10%) folds and iteratively increased from 0.1 to 1 (100%) of the training data in the target domain, as shown in Figure 3. To study the influence of the UGM selection, each split is performed 10 times. Due to the deterministic training of FESR, the error bars shown in the results describe the influence of the UGM selection, i.e., the variance over the 10 repetitions. Besides the comparison with the target-domain model, the proposed IDNNRep retraining approach is also evaluated against OSC, in which one orthogonal component is removed as introduced in [38]. The data used for the OSC algorithms are 100% of the base domain training data. DS was not considered because Calibration 1 and Calibration 2 differ in the gas compositions of the UGM and the target gas concentrations. Since DS requires corresponding transfer samples measured under both conditions, it is not applicable in this experimental setup. This highlights a key limitation of DS, whereas neither OSC nor the proposed IDNNRep retraining requires such identical calibration samples.

The novel approach allows adjustments to be traced directly back to the extracted features, without the need for additional XAI (Explainable AI) techniques [39]. This provides a more transparent view of the influence of individual features on the model adaptation. To understand the influence of each feature, it is necessary to examine how the concentration quantifications are computed. The specific concentration c is calculated from the extracted features as follows:

(9) $$c=\left[\begin{array}{ccc} \beta_{1} & \cdots & \beta_{n} \end{array}\right](W_{FS\;}\vec{z})+\;\beta_{0}$$

Equation (11) describes the weighting of each selected feature for the estimation of the gas concentration. Since the transfer learning only retrains the FS and PLSR layers, changes in the layer weights directly influence the importance of each feature for gas concentration quantification. For this purpose, changes in the PLSR components and the FS layer are combined to return the extracted feature to its specific value. The changes in the feature weights $d_{feat}$ are calculated as shown in (10), with ${{W^{\prime }}_{FS}\;\beta^{\prime }}_{PLSR}$ as the new weights of the features. After the calculation, the $d_{feat}$ is rescaled, to show the relative changes as described in (11):

(10) $$d_{feat}=abs({({\;W}_{FS}\;\beta }_{PLSR})-{({\;W^{\prime }}_{FS}\;\beta^{\prime }}_{PLSR}))$$

(11) $$d_{feat\_scale}=\frac{d_{feat}-min(d_{feat})}{max(d_{feat})-min(d_{feat})}$$

This allows us to analyze the change in feature weighting for each feature individually. The detection of relevant features changes is discussed in the results chapter.

## 3. Results

**Figure 6:**
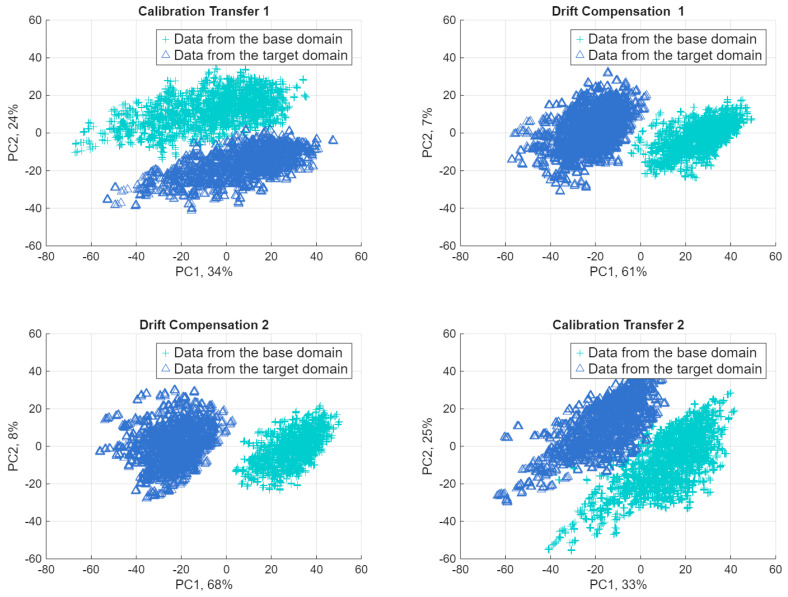
Scatter plot of the standardized principal component analysis for the different domains of the features.

Before presenting the results of the novel interpretable and model-based calibration transfer, a brief exploration of the data is provided. The domain shifts included in this study are shown in Figure 2. This study presents two types of domain shifts in MOS gas sensing: batch-to-batch and drift. To demonstrate the significant shift in the data distribution, a standardized PCA [40] is applied to the mean and slope features of the one-second intervals and shown in Figure 6. The PCA plots show a significant shift in the sensor data for all four domain shifts. The drift effect is reflected by a significant shift along the first principal component (PC), while the batch shift results in a shift primarily along the 2nd PC for the sensors before field operation and in both PCs for the sensors after field operation. The stronger domain shift is observed for drift, which could make it more difficult to adapt the model for drift compensation.

The dataset comprises multiple domain-shift scenarios originating from both calibration transfer between different sensor batches and irreversible sensor drift. Furthermore, the included UGMs comprise eight VOCs, resulting in complex nonlinear sensor response characteristics and a challenging calibration task. As illustrated by the PCA analysis (Figure 6), the source and target domains exhibit a clear separation with varying cluster distances, indicating domain shifts of different severity and providing representative benchmark scenarios for evaluating the proposed adaptation method.

For the demonstration of the novel introduced method, the data are separated as follows for the training and test data sets, as seen in Table 2. The novel approach is demonstrated for two different gases, acetone and toluene, which are included in all measurements. The calibration models are assessed with the metrics root mean square error (RMSE) and coefficient of determination (R^2^). RMSE provides a quantitative measure of prediction error, while R^2^ characterizes the model’s goodness-of-fit and its ability to describe the variability of the unseen domain.

### 3.1. R^2^ Results

While RMSE provides an absolute measure of prediction error [41], R^2^ provides insight into how well the model’s variability explains the variability in the reference data after transfer [42]. By considering both metrics together, a more comprehensive assessment of the calibration transfer performance can be achieved. Before analyzing the RMSE, the R^2^ is first analyzed to confirm that the model successfully captures the underlying relationship between the input features and the target variable. Subsequently, the RMSE is evaluated to quantify the magnitude of the prediction error and the resulting calibration accuracy (n RMSE: normalized on the maximum concentration). A comparison with the R^2^ value of the initial model in the target domain is omitted, as the initial model yielded poor predictive performance across all shift scenarios. Consequently, both calibration transfer approaches, IDNNRep retraining and the target-domain model, significantly outperform the initial model.

**Figure 7:**
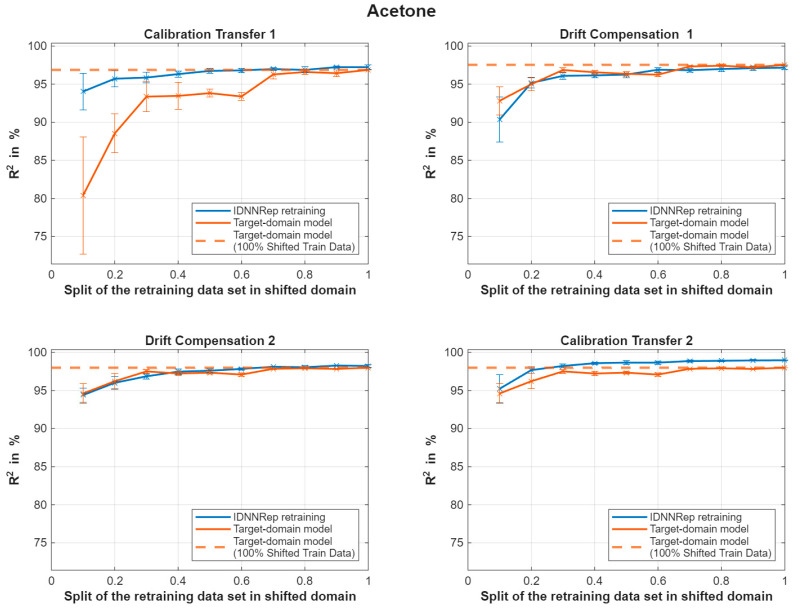
Comparison of different model R^2^ values of the retrained and the target-domain model regarding acetone for the four domain shifts.

Figure 7 shows the R^2^ performance of the IDNNRep retraining and the target-domain model across the four domain-shift scenarios for acetone quantification. For acetone both approaches achieve generally high R^2^ values between 90% and 98%, across all four domain-shift scenarios, indicating that both models explain most of the variance and that the calibration model quantifications correlate strongly with the target. For Calibration Transfer 1, the R^2^ value of the target-domain model starts comparably low at 80% for a 0.1 split of the UGMs with a high standard deviation. The IDNNRep starts at 94% in the Calibration Transfer 1 scenario, showing a significant improvement vs. training from scratch. For all domain-shift scenarios and both approaches, the R^2^ value increases nearly monotonically with the number of transfer samples and converges to the R^2^ value reached with all transfer samples from the target domain.

**Figure 8:**
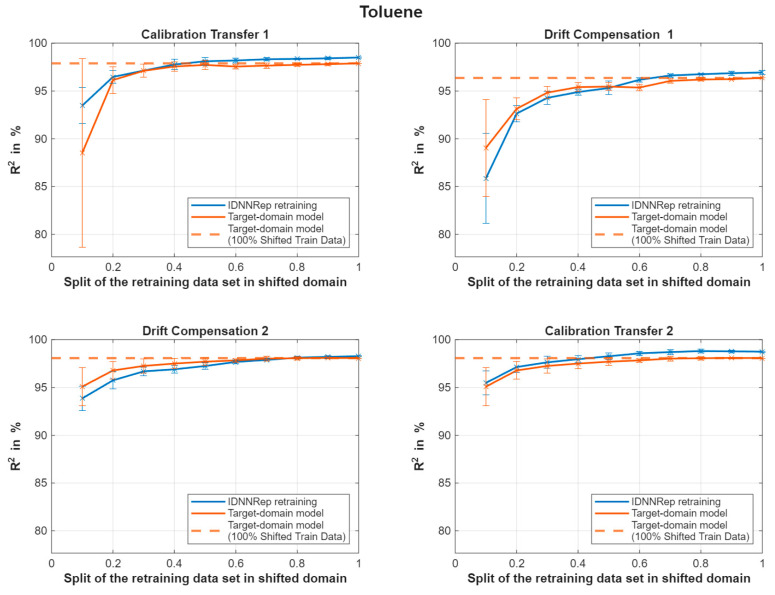
Comparison of different model R^2^ values of the retrained and the target-domain model regarding toluene for the four domain shifts.

The same comparison can be made for the toluene estimation (see Figure 8). The R^2^ values for the toluene estimation follow a similar trend to those for the acetone estimation. Both approaches yield similarly high R^2^ values (95%) across all scenarios when the data split is greater than 0.1. The upward trends indicate that both models effectively adapt to domain shifts, achieving good generalization with a limited fraction of new data. However, the target-domain model exhibits slightly lower or the same R^2^ values, particularly evident in Calibration Transfer 1 for data splits below 0.4. Despite this, both methods also showed weak performance for the Drift Compensation 1 domain shift at small data splits (<0.3). However, especially during training with a higher number of transfer samples, both models show good performance, with R^2^ values ranging from 92% to 98%, demonstrating strong explained variance in the calibration model for gas estimation across all domain shifts. To summarize, the models for both gases converge quickly toward the target-domain model trained with all transfer sample baselines, with negligible differences between them. This suggests both approaches are comparably efficient once a moderate amount of calibration transfer data is available. Also, the error bars diminish rapidly as retraining data increases, signifying good reproducibility and model stability for data splits with approx. 0.3–0.4 of the full target-domain dataset. Note, however, that this corresponds to 60 to 80 gas mixtures, already representing a high effort. Performance close to full-data retraining can be obtained with less than half of the available target-domain data, highlighting the efficiency and robustness of both transfer strategies. Transfer learning for the IDNNRep can reduce the amount of training data required significantly, with 20 or 40 gas mixtures being required to achieve a performance similar to retraining the full model for both gases and all domain-shift scenarios.

### 3.2. RMSE Results

After the R^2^ values proved that the model is capable of capturing the relationship between input and target, Table 3 summarizes the RMSE and the normalized RMSE (normalized regarding the maximum of the corresponding concentration range) of the initial FESR calibration model under conditions of significant domain shift. The model demonstrates robust performance in the presence of batch-related shifts, yielding prediction errors of 70.5 ppb for Calibration Transfer 1 and 73.7 ppb for Calibration Transfer 2 for the acetone estimation. In contrast, temporal domain shifts substantially degrade model performance, with errors increasing to 266.9 ppb (Drift Compensation 1) and 300.1 ppb (Drift Compensation 2) for acetone. Note that the observed RMSE after the domain shift of Drift Compensation 2 for acetone actually exceeds the studied gas concentration range, indicating very poor performance of the initial calibration model on previously unseen data.

Table 3 also shows that applying OSC only slightly affects the base-domain performance, increasing the RMSE by approximately 1–5 ppb across all scenarios. By contrast, its influence on the target-domain performance strongly depends on the underlying domain shift. For the drift compensation scenarios, OSC considerably reduces the RMSE, for example from 266.9 ppb to 237.4 ppb for acetone (Drift Compensation 1), from 300.1 ppb to 119.8 ppb for acetone (Drift Compensation 2), and from 151.9 ppb to 105.3 ppb for toluene (Drift Compensation 1). However, for the calibration transfer scenarios, OSC provides only limited improvements or even degrades the prediction accuracy, e.g., increasing the RMSE from 70.5 ppb to 108.1 ppb for acetone (Calibration Transfer 1) and from 73.7 ppb to 93.1 ppb for acetone (Calibration Transfer 2). These results indicate that OSC is mainly beneficial at drift compensation, whereas it is less effective for calibration transfer scenarios.

**Figure 9:**
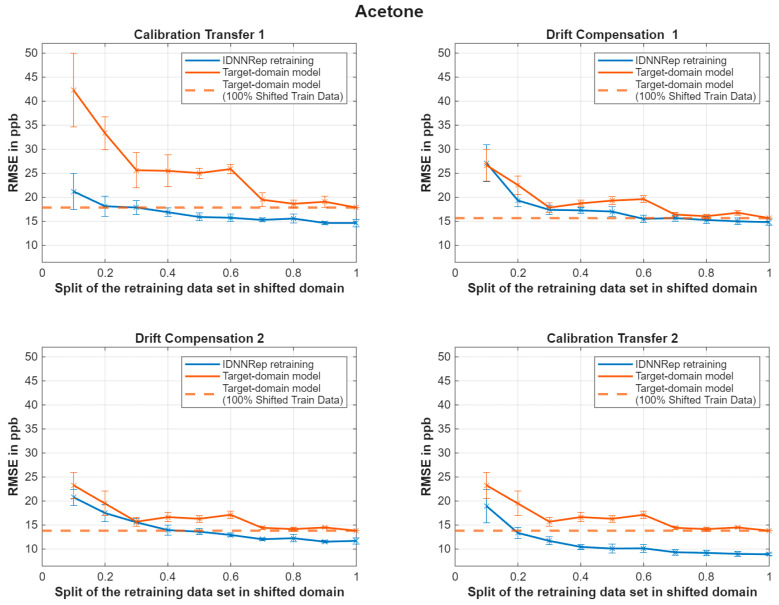
Comparison of the different RMSEs of the retrained and the target-domain model regarding acetone for the four domain shifts.

The novel approach allows the fine-tuning of this initial calibration model to adapt to the target domain. Figure 9 presents the RMSE results for acetone quantification using the retrained IDNNRep and the target-domain FESR model, respectively, for different proportions of target-domain training data. Performance is evaluated under the four pre-defined domain-shift scenarios. Across all domain-shift scenarios, the retrained IDNNRep shows a consistent decrease in RMSE as the number of transfer samples from the novel domain increases. This is observed for both types of domain shifts, the batch-related shifts (Calibration Transfer 1, Calibration Transfer 2) and temporal shifts (Drift Compensation 1, Drift Compensation 2). In the Calibration Transfer shift scenarios, RMSE decreases from 21.2 ppb to 14.6 ppb for Calibration Transfer 1 and from 18.9 ppb to 8.9 ppb for Calibration Transfer 2. Notably, Calibration Transfer 2 consistently yields the lowest errors across all splits, indicating that this batch shift is either less severe or better aligned with the learned representation after transfer. In the drift scenarios, a similar improvement is observed. In Drift Compensation 1, the RMSE decreases from 27.0 ppb to 14.8 ppb, and in Drift Compensation 2, it decreases from 20.7 ppb to 11.7 ppb. Drift Compensation 1 consistently shows a higher RMSE than Drift Compensation 2, suggesting that Drift Compensation 1 involves a more challenging temporal-domain shift. Importantly, the model achieves significant performance gains even with a small number of transfer samples (split 0.1–0.3) across all scenarios, compared to the initial model.

The reduced FESR model also shows improved performance as the transfer sample increases from the novel domain. Under batch shift conditions, the target-domain model exhibits a higher RMSE in Calibration Transfer 1 with a small number of UGMs (42.3 ppb at split 0.1). Although the RMSE decreases to 17.8 ppb, it remains consistently higher than that of IDNNRep. In Calibration Transfer 2, the RMSE is lower, starting at 23.2 ppb and decreasing to 13.8 ppb. For the time-related domain shift, the model estimation RMSE is reduced from 26.7 ppb to 15.6 ppb for Drift Compensation 1 and from 23.3 ppb to 13.8 ppb for Drift Compensation 2. The difference between the IDNNRep and the target-domain model is significantly smaller for time-related domain shifts in predicting acetone. Figure 9 also shows that the improvement in acetone estimation with the full number of transfer samples is greater for batch-related domain shifts. In addition, the IDNNRep outperforms the target-domain model in every domain shift, even if the target-domain model is trained on all available transfer samples.

**Figure 10:**
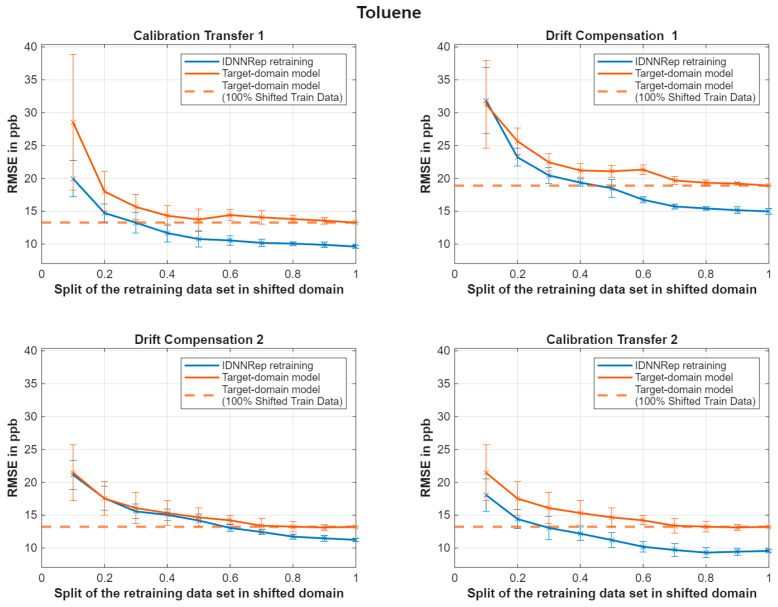
Comparison of the different RMSEs of the retrained and the target-domain model regarding toluene for the four domain shifts.

The same comparison for toluene is shown in Table 3 and Figure 10. The initial model (no transfer) exhibits substantially higher RMSE values across all scenarios (122.3–158.8 ppb), highlighting the necessity of transfer learning. The same trend as observed for the acetone estimation is also shown in Figure 10 for the toluene concentration quantifications. Under batch shift conditions, both models improve substantially with increasing split, with Calibration Transfer 1 representing the more challenging scenario. IDNNRep reduces RMSE from 20.0 ppb to 9.6 ppb in Calibration Transfer 1 and from 18.1 ppb to 9.6 ppb in Calibration Transfer 2, while the target-domain model decreases from 28.5 ppb to 13.3 ppb in Calibration Transfer 1 and from 21.4 ppb to 13.2 ppb in Calibration Transfer 2. For the temporal shifts, the IDNNRep improves from 31.9 ppb to 15.0 ppb, whereas the target-domain model reaches 18.9 ppb in Drift Compensation 1. In the Drift Compensation 2 domain shift, the IDNNRep improves from 21.1 ppb to 11.2 ppb, and the target-domain model from 21.4 ppb to 13.2 ppb. While both models clearly improve the performance, the IDNNRep maintains a clear performance advantage especially for a higher number of transfer samples. For a small amount of transfer sample, the IDNNRep matches (time-related) or even outperforms (batch-related) the RMSE of the target-domain model. By increasing the number of UGMs the IDNNRep outperforms the target-domain model across all domain shifts for the toluene estimation.

The proposed approach reduces the RMSE using only a small amount of retraining data (split: 0.1), achieving a reduction of up to 93% for Drift Compensation 2 on acetone, decreasing the RMSE from 300.1 ppb for the initial model to 21.1 ppb. Furthermore, the proposed IDNNRep approach consistently outperforms OSC on the presented dataset, although OSC also improves the transferability of the initial calibration model. Compared with OSC, IDNNRep achieves an additional RMSE reduction of up to 89.0% for Drift Compensation 1 while requiring only 10% of the target-domain data for retraining. For both gases, the standard deviation of a small number of UGMs for the target-domain model is typically higher than that of the IDNNRep, except for acetone in Calibration Transfer 2. Increasing the transfer sample reduces the influence of random UGM splitting, and the standard deviation between models decreases accordingly.

### 3.3. Interpretable Calibration Transfer

**Figure 11:**
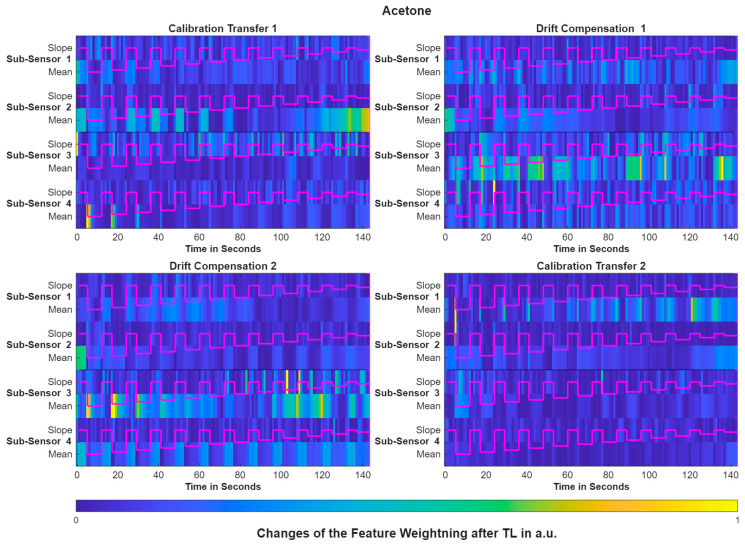
Interpretable changes after the calibration transfer for acetone quantification.

**Figure 12:**
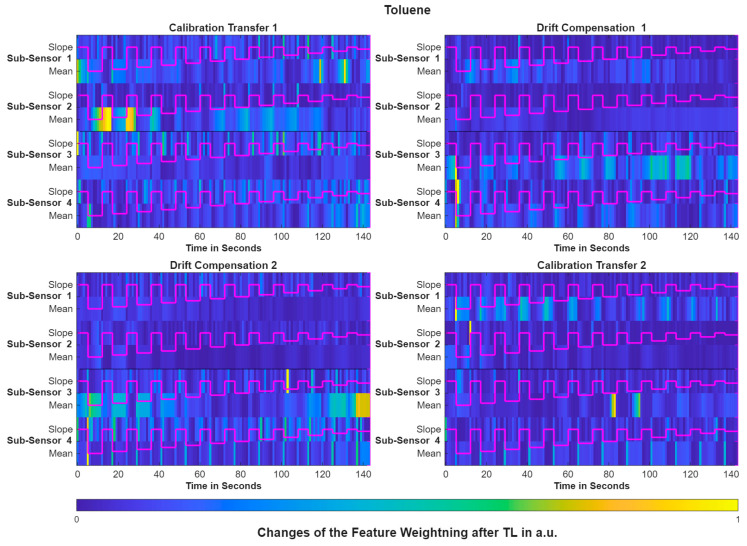
Interpretable changes after the calibration transfer for toluene quantification.

To gain insight into how sensor characteristics or environmental factors influence model adaptation, an interpretation of the calibration transfer can describe how calibration models adapt to new domains. This transparency enhances diagnostic understanding and model robustness, which are essential for deploying sensor systems in real-world applications. Figure 11 and Figure 12 demonstrate the changes in the weighting of the mean and slope features extracted from the four sub-sensors of the gas sensor for the four domain-shifts for the quantification of acetone and toluene, respectively. The color intensity indicates the magnitude of the change in the extracted features, ranging from zero to one. By combining feature analysis with the TC, it becomes possible to identify which phase in the TC each feature is associated with. Figure 11 highlights that the majority of calibration transfer adjustments for acetone quantification across all domain-shifts occur within the mean features and this primarily for sub-sensors 2 and 3. While adjustments are seen across the full range for most domain-shift scenarios, Calibration Transfer 1 shows strong adjustments at the beginning and end of the TC for sub-sensor 2 and also in the beginning of the TC for sub-sensor 4. Sub-sensor 1 remains largely stable throughout the transfer process, except for Calibration Transfer 2, which probably means that it contributes only little information for acetone quantification. Sub-sensor 4 shows some adaptation of the mean features, mainly for Calibration Transfer 1. Sub-sensor 3 is the only sub-sensor which exhibits significant adaptation for the slope features, basically across all domain-shift scenarios.

For the toluene, the most significant adaptations are observed in the mean values of sub-sensor 2 and 3, as illustrated in Figure 12, which was also observed for acetone. This indicates that the multi-sensor character is an important aspect for the performance of the overall sensor system. Sub-sensor 1 overall shows the least changes, indicating that it contributes only little to toluene quantification. Slope adaptions occur primarily at transitions between two temperature phases, which is consistent with the Sauerwald–Baur model for temperature-cycled operation [43].

## 4. Discussion

This paper describes a novel approach enabling calibration transfer for interpretable FESR models for a multi-layer MOS gas sensor operated in TCO mode in complex gas mixtures with up to eight independent variables demonstrated for quantification of acetone and toluene, respectively. The novel approach was tested on laboratory data sets that include several domain-shift scenarios covering both sensor batch changes as well as sensor drift over time. The novel approach was tested across four significant domain-shift scenarios and compared against classical methods (OSC). The results demonstrate that the novel approach enables model-based calibration transfer with significant reduction in the calibration effort compared to recalibration from scratch and outperforms the OSC technique for the selected domain shifts. For a small number of transfer samples, the model-based calibration transfer outperforms training a new FESR model from scratch. Remarkably, the transfer learning approach consistently achieves a lower RMSE than a model trained solely for and with data from the novel domain. Additionally, in some scenarios the IDNNRep even outperforms the target-domain model trained on the full data. This demonstrated that the IDNNRep increases the dimensionality by including all features for the retraining process. For both gases the drift-related domain shifts appear to be more challenging for the calibration transfer than the batch-related ones. This is evidenced by the higher loss of performance when only a limited number of transfer samples (UGMs) are available for drift compensation. In addition to achieving a low RMSE, the proposed approach effectively adapts the initial model to the new domain, as evidenced by the significant improvement in R^2^. This demonstrates the method’s capability to maintain both predictive accuracy and model consistency across varying domain conditions. The high R^2^ value for the target-domain model and the IDNNRep proves the efficient and robust model building of the FESR methods trained with a reduced number of UGMs. Additionally, the variance caused by the random selection of the transfer sample is reduced by using transfer learning to adapt an initial model to a novel domain.

On the one hand, these results demonstrate the robustness of the proposed interpretable calibration transfer approach by effectively reusing knowledge learned in the base domain. On the other hand, they highlight the importance of selecting suitable UGMs for calibration transfer, which remains an open research challenge and warrants further investigation. Since both the initial model training and the IDNNRep retraining are nearly deterministic, the variability introduced by the learning algorithm itself is negligible, as indicated by the near-zero error bars for larger transfer splits. Consequently, the error bars reported in the RMSE figures primarily reflect the influence of the transfer UGM selection rather than the training process itself, emphasizing the critical role of representative transfer samples in MOS gas sensor calibration.

While this study demonstrated the successful implementation of model-based calibration transfer, it did not include a detailed hyperparameter search for transfer learning. This search could further improve the performance of the IDNNRep retraining. Besides the performance gains of the novel approach, it also demonstrates the interpretable nature of FESR model calibration transfer, without further usage of XAI. This enables a better understanding of the changes leading to domain shift and necessitating retraining for different sensing layers and gases. In particular, the identification of features which are highly affected by domain shift is possible. By understanding the adaptation of features caused by domain shift, the interpretation allows us to identify robust features for future model building and offers the potential to understand the decrease in the calibration model performance. Additionally, in-depth interpretation of the calibration model allows identification of relevant phases of the TC, offering the potential to optimize the TC for different target gases and applications. Since the data investigated were collected in a controlled lab environment, the representativeness of the dataset for large-scale industrial deployment remains to be validated using real-world data.

## 5. Conclusions and Outlook

In conclusion, the IDNNRep can be used to enable transfer learning for interpretable calibration models, adapting them to unseen domains with a low amount of transfer samples. The novel approach was able to quantify the acetone concentration with an RMSE of 18.0–28.0 ppb (R^2^: 94–98%) and the toluene concentration with an RMSE of 18.0–32.0 ppb (R^2^: 86–95%) across the four different domain-shift scenarios, by only using 20 transfer samples (split: 0.1). The results demonstrate the efficient usage of model-based calibration transfer for interpretable FESR calibration models and the performance gain compared to OSC. In addition to the efficient implementation offered by the IDNNRep, this contribution extends the IDNNRep potential to adaptation of FESR models to new domains, an essential characteristic for field operation. This capability is particularly important for field operation, where changing environmental conditions and sensor replacement may require adaptation of the calibration model. Future work should also investigate in-field applications in which transfer learning is directly applied under real-world operating conditions.

By reusing the initial calibration model, the proposed approach reduces the number of transfer samples required for recalibration. Furthermore, a potential limitation of the proposed method is its applicability to domain shifts that exceed those represented in the available datasets, such as previously unseen gas compositions or extreme changes in operating conditions. Investigating the robustness of the proposed approach under such scenarios represents an important direction for future work.

Since the model developed in this study was optimized primarily for performance in the base domain, future work should also focus on improving its robustness to domain shifts. In particular, the standardization procedure, whose parameters were estimated exclusively from the base domain, may limit the model’s ability to adapt during retraining. Future research should therefore investigate (a) allowing the standardization layer to be fine-tuned within the IDNNRep framework and (b) recalculating the standardization parameters using representative samples from the target domain. Both approaches have the potential to further improve model performance under target-domain conditions.

The knowledge gained through the interpretation of the weight changes can be used in future work to gain sensor- and gas-specific insights into the calibration process, thereby optimizing the complex process for sensor system development, including sensor selection, optimized operating modes, and the selection of calibration points. Furthermore, future work should investigate the relationship between the learned weight changes and the underlying physicochemical processes within the sensor, such as sensing-layer degradation or changes in reaction kinetics. Establishing this relationship could further improve the interpretability of the proposed approach and provide valuable insights into the mechanisms driving calibration transfer and sensor drift. Further research could also investigate advanced FESR models that extract features beyond simple mean-slope calculations, including varying time segmentation for feature calculation [44]. Such approaches may enable a more comprehensive characterization of the underlying signals. In addition, future work could build upon the insights from the training process in this study to systematically identify features that are both robust and reliable, thereby improving the overall performance and generalizability of the calibration models, while still allowing for further improvement through model-based calibration transfer for the interpretable FESR models. Since this novel approach is not limited to the transfer of MOS gas sensor calibration models, future research could also address use-cases from different applications, e.g., industrial condition monitoring using multi-modal sensor systems [45] or other fields where changing conditions decrease model performance.

## Figures and Tables

**Table 1:** Concentration ranges for all target gases are divided into the first and second calibration within the dataset.

	First Calibration		Second Calibration	
Volatiles	Min.	Max.	Min.	Max.
Acetone	1 ppb	300 ppb	1 ppb	300 ppb
Carbon monoxide	100 ppb	2000 ppb	-	-
Ethanol	1 ppb	300 ppb	1 ppb	300 ppb
Ethyl acetate	1 ppb	300 ppb	-	-
Formaldehyde	1 ppb	300 ppb	1 ppb	300 ppb
Hydrogen	400 ppb	1900 ppb	400 ppb	1900 ppb
Toluene	1 ppb	300 ppb	1 ppb	300 ppb
Rel. humidity @20 °C	25% RH	70% RH	25% RH	75% RH

**Table 2:** Data splitting for the four different domain-shift scenarios.

Scenario	Model	Train UGM	Test UGM
**Calibration Transfer 1**	Initial	**Base train data**: UGM Batch 1 in Initial Calibration	**Target test data:** Batch 2 in Initial Calibration
	Target-domain	**Tuning data**: 0.1 .. 1 split of Batch 2 in Initial Calibration	
	IDNNRep		
Drift Compensation **1**	Initial	**Base train data**: Batch 1 in Initial Calibration	**Target test data:** Batch 1 in Recalibration
	Target-domain	**Tuning data**: 0.1 .. 1 split of Batch **1** in Recalibration	
	IDNNRep		
Drift Compensation **2**	Initial	**Base train data**: Batch 2 in Initial Calibration	**Target test data:** Batch 2 in Recalibration
	Target-domain	**Tuning data**: 0.1 .. 1 split of Batch 2 in Recalibration	
	IDNNRep		
**Calibration Transfer 2**	Initial	**Base train data**: Batch 1 in Recalibration	**Target test data:** Batch 2 in Recalibration
	Target-domain	***Tuning data:*** *0.1 .. 1 split of Batch 2 in Recalibration*	
	IDNNRep		

**Table 3:** Initial FESR model performance for the different test scenarios for acetone and toluene, including orthogonal signal correction.

	Acetone				Toluene			
	RMSE (nRMSE)				RMSE (nRMSE)			
	Base Domain		Target Domain		Base Domain		Target Domain	
	No OSC	OSC	No OSC	OSC	No OSC	OSC	No OSC	OSC
**Calibration Transfer 1**	16.6 ppb **(5.5%)**	21.1 ppb **(7.0%)**	70.5 ppb **(23.5%)**	108.1 ppb **(36.0%)**	13.6 ppb **(4.5%)**	15.2 ppb **(5.1%)**	122.3 ppb **(40.7%)**	112.7 ppb **(37.6%)**
**Drift Compensation 1**	16.6 ppb **(5.5%)**	20.9 ppb **(6.9%)**	266.9 ppb **(88.9%)**	237.4 ppb **(79.1%)**	13.6 ppb **(4.5%)**	14.7 ppb **(4.9%)**	151.9 ppb **(50.6%)**	105.3 ppb **(35.1%)**
**Drift Compensation 2**	12.9 ppb **(4.3%)**	14.7 ppb **(4.9%)**	300.1 ppb **(100%)**	119.8 ppb **(39.9%)**	8.3 ppb **(2.7%)**	8.5 ppb **(2.8%)**	114.9 ppb **(48.3%)**	106.4 ppb **(35.5%)**
**Calibration Transfer 2**	10.8 ppb **(3.6%)**	11.9 ppb **(4.0%)**	73.7 ppb **(24.5%)**	93.1 ppb **(31.0%)**	13.8 ppb **(4.6%)**	15.6 ppb **(5.2%)**	158.8 ppb **(52.9%)**	134.0 ppb **(44.6%)**

**Figure A1:**
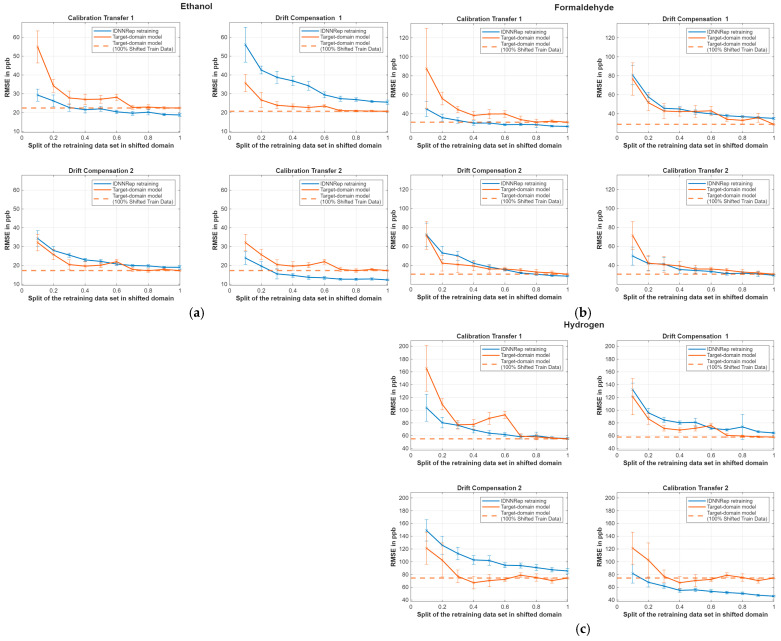
Comparison of the different RMSEs of the retrained and the target-domain model for the four domain shifts. (**a**) Ethanol; (**b**) formaldehyde; (**c**) hydrogen.

**Figure A2:**
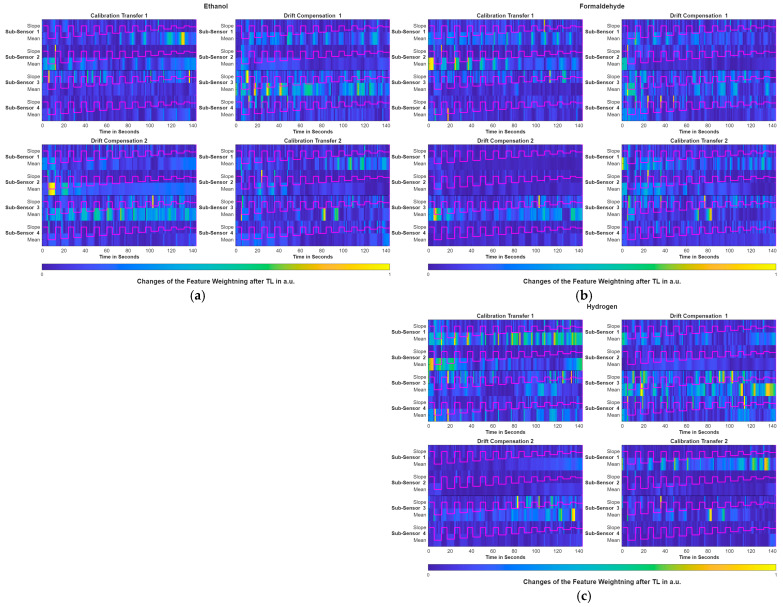
Interpretable changes after the calibration transfer for quantification. (**a**) Ethanol; (**b**) formaldehyde; (**c**) hydrogen.

## Data Availability

The datasets used in this study are publicly available. Detailed information on the sources of these datasets can be found in the corresponding sections of this paper.

## Abbreviations

The following abbreviations are used in this manuscript:

DNN	Deep Neural Network
DS	Direct Standardization
FE	Feature Extraction
FESR	Feature Extraction, Feature Selection und Regression
GDB	Global Disease Burden
GMA	Gas Mixtures Analysis
IAQ	Indoor Air Quality
IDNNRep	Interpretable Deep Neural Network Representation
ML	Machine Learning
MOS	Metal Oxide Semiconductor
OSC	Orthogonal Signal Correction
PCA	Principle Component Analysis
PLSR	Partial Least Squares Regression
RFE-LSR	Recursive Feature Elimination—Least Squares Regression
RMSE	Root Mean Square Error
TC	Temperature-Cycled
TCO	Temperature-Cycled Operation
TL	Transfer Learning
UGM	Unique Gas Mixtures
VOC	Volatile Organic Compounds
XAI	Explainable Artificial Intelligence

## References

[1] Murray C.J., Aravkin A.Y., Zheng P., Abbafati C., Abbas K.M., Abbasi-Kangevari M., Abd-Allah F., Abdelalim A., Abdollahi M., Abdollahpour I. (2020). Global Burden of 87 Risk Factors in 204 Countries and Territories, 1990–2019: A Systematic Analysis for the Global Burden of Disease Study 2019. Lancet.

[2] Williams M.L., Olomukoro A.A., Emmons R.V., Godage N.H., Gionfriddo E. (2023). Matrix Effects Demystified: Strategies for Resolving Challenges in Analytical Separations of Complex Samples. J. Sep. Sci..

[3] Fine G.F., Cavanagh L.M., Afonja A., Binions R. (2010). Metal Oxide Semi-Conductor Gas Sensors in Environmental Monitoring. Sensors.

[4] Wolkoff P., Nielsen G.D. (2001). Organic Compounds in Indoor Air—Their Relevance for Perceived Indoor Air Quality?. Atmos. Environ..

[5] WHO Guidelines for Indoor Air Quality: Selected Pollutants. https://www.who.int/publications/i/item/9789289002134.

[6] Kumar P., Morawska L., Martani C., Biskos G., Neophytou M., Di Sabatino S., Bell M., Norford L., Britter R. (2015). The Rise of Low-Cost Sensing for Managing Air Pollution in Cities. Environ. Int..

[7] Baur T., Amann J., Schultealbert C., Schütze A. (2021). Field Study of Metal Oxide Semiconductor Gas Sensors in Temperature Cycled Operation for Selective VOC Monitoring in Indoor Air. Atmosphere.

[8] Borrás E., Herrmann H., Kalberer M., Muñoz A., Mutzel A., Vera T., Wenger J., Doussin J.-F., Fuchs H., Kiendler-Scharr A., Seakins P., Wenger J. (2023). Sampling for Offline Analysis. A Practical Guide to Atmospheric Simulation Chambers.

[9] Dey A. (2018). Semiconductor Metal Oxide Gas Sensors: A Review. Mater. Sci. Eng. B.

[10] Singh S., S S., Varma P., Sreelekha G., Adak C., Shukla R.P., Kamble V.B. (2024). Metal Oxide-Based Gas Sensor Array for VOCs Determination in Complex Mixtures Using Machine Learning. Microchim Acta.

[11] Gramm A., Schütze A. (2003). High Performance Solvent Vapor Identification with a Two Sensor Array Using Temperature Cycling and Pattern Classification. Sens. Actuators B Chem..

[12] Schütze A., Sauerwald T., Jaaniso R., Tan O.K. (2020). Dynamic Operation of Semiconductor Sensors. Semiconductor Gas Sensors.

[13] Robin Y., Amann J., Schneider T., Schütze A., Bur C. (2023). Comparison of Transfer Learning and Established Calibration Transfer Methods for Metal Oxide Semiconductor Gas Sensors. Atmosphere.

[14] Robin Y., Amann J., Baur T., Goodarzi P., Schultealbert C., Schneider T., Schütze A. (2021). High-Performance VOC Quantification for IAQ Monitoring Using Advanced Sensor Systems and Deep Learning. Atmosphere.

[15] Arendes D., Amann J., Tessier C., Brieger O., Schütze A., Bur C. (2023). Qualification and Optimisation of a Gas Mixing Apparatus for Complex Trace Gas Mixtures. TM-Tech. Mess..

[16] Krutzler C., Unger A., Marhold H., Fricke T., Conrad T., Schütze A. (2012). Influence of MOS Gas-Sensor Production Tolerances on Pattern Recognition Techniques in Electronic Noses. IEEE Trans. Instrum. Meas..

[17] Schultealbert C., Diener R., Amann J., Baur T., Schütze A., Sauerwald T. (2020). Differential Scanning Calorimetry on Micro Hotplates for Temperature Calibration and Mass Quantification. TM-Tech. Mess..

[18] Romain A.C., Nicolas J. (2010). Long Term Stability of Metal Oxide-Based Gas Sensors for e-Nose Environmental Applications: An Overview. Sens. Actuators B Chem..

[19] Dennler N., Rastogi S., Fonollosa J., van Schaik A., Schmuker M. (2022). Drift in a Popular Metal Oxide Sensor Dataset Reveals Limitations for Gas Classification Benchmarks. Sens. Actuators B Chem..

[20] Li R., Li Z., Kofi B.A., Sun J., He Y., Jiao M. (2026). A Comprehensive Review of Algorithms for Drift Compensation in Metal Oxide Semiconductor Gas Sensor Arrays. Chemosensors.

[21] Schneider T., Helwig N., Schütze A. Automatic feature extraction and selection for condition monitoring and related datasets. Proceedings of the 2018 IEEE International Instrumentation and Measurement Technology Conference (I2MTC).

[22] Fonollosa J., Neftci E., Huerta R., Marco S. (2015). Evaluation of Calibration Transfer Strategies between Metal Oxide Gas Sensor Arrays. Procedia Eng..

[23] Fonollosa J., Fernández L., Gutiérrez-Gálvez A., Huerta R., Marco S. (2016). Calibration Transfer and Drift Counteraction in Chemical Sensor Arrays Using Direct Standardization. Sens. Actuators B Chem..

[24] Robin Y., Amann J., Goodarzi P., Schneider T., Schütze A., Bur C. (2022). Deep Learning Based Calibration Time Reduction for MOS Gas Sensors with Transfer Learning. Atmosphere.

[25] Wolfrum E.J., Meglen R.M., Peterson D., Sluiter J. (2006). Calibration Transfer Among Sensor Arrays Designed for Monitoring Volatile Organic Compounds in Indoor Air Quality. IEEE Sens. J..

[26] Yan K., Zhang D. (2016). Calibration Transfer and Drift Compensation of e-Noses via Coupled Task Learning. Sens. Actuators B Chem..

[27] Li J., Yuan Z., Guo Z., Meng F. (2026). An Attention-Enhanced Deep Transfer Learning Method for Few-Shot Calibration of Semiconductor Gas Sensors. Sens. Actuators B Chem..

[28] Buhrmester V., Münch D., Arens M. (2021). Analysis of Explainers of Black Box Deep Neural Networks for Computer Vision: A Survey. Mach. Learn. Knowl. Extr..

[29] Arendes D., Robin Y., Amann J., Petto A., Schütze A., Bur C. Transfer Learning Between Two Different Datasets of MOS Gas Sensors. Proceedings of the 2024 IEEE International Symposium on Olfaction and Electronic Nose (ISOEN).

[30] Goodarzi P., Schütze A., Schneider T. (2025). Domain shifts in industrial condition monitoring: A comparative analysis of automated machine learning models. J. Sens. Sens. Syst..

[31] Petry J., Schu D., Mertin T., Arendes D. (2024). Optimisation of Convolutional Neural Networks for MOS Gas Sensors. Proceedings of the 17th Dresdner Sensor-Symposium, Dresden, Germany, 25–27 November 2024.

[32] Schauer J., Goodarzi P., Schütze A., Schneider T. (2025). Efficient hardware implementation of interpretable machine learning based on deep neural network representations for sensor data processing. J. Sens. Sens. Syst..

[33] Schauer J., Goodarzi P., Morsch J., Schütze A. (2025). A Performance Study of Deep Neural Network Representations of Interpretable ML on Edge Devices with AI Accelerators. Sensors.

[34] Arendes D., Amann J.F., Schütze A., Bur C. (2025). Lab Calibrations of MOS Gas Sensors: Dataset to Investigate Drift Compensation.

[35] (2017). ZeMA-gGmbh LMT-ML-Toolbox. GitHub Repository.

[36] Chen X., Jeong J.C. Enhanced Recursive Feature Elimination. Proceedings of the Sixth International Conference on Machine Learning and Applications (ICMLA 2007).

[37] Geladi P., Kowalski B.R. (1986). Partial Least-Squares Regression: A Tutorial. Anal. Chim. Acta.

[38] Padilla M., Perera A., Montoliu I., Chaudry A., Persaud K., Marco S. (2010). Drift Compensation of Gas Sensor Array Data by Orthogonal Signal Correction. Chemom. Intell. Lab. Syst..

[39] Dwivedi R., Dave D., Naik H., Singhal S., Omer R., Patel P., Qian B., Wen Z., Shah T., Morgan G. (2023). Explainable AI (XAI): Core Ideas, Techniques, and Solutions. ACM Comput. Surv..

[40] Greenacre M., Groenen P.J.F., Hastie T., d’Enza A.I., Markos A., Tuzhilina E. (2022). Principal component analysis. Nat. Rev. Methods Prim..

[41] Tatachar A.V. (2021). Comparative Assessment of Regression Models Based on Model Evaluation Metrics. Int. Res. J. Eng. Technol. IRJET.

[42] Chicco D., Warrens M.J., Jurman G. (2021). The Coefficient of Determination R-Squared Is More Informative than SMAPE, MAE, MAPE, MSE and RMSE in Regression Analysis Evaluation. PeerJ Comput. Sci..

[43] Schultealbert C., Baur T., Schütze A., Böttcher S., Sauerwald T. (2017). A Novel Approach towards Calibrated Measurement of Trace Gases Using Metal Oxide Semiconductor Sensors. Sens. Actuators B Chem..

[44] Fulcher B.D. (2018). Feature-Based Time-Series Analysis. Feature Engineering for Machine Learning and Data Analytics.

[45] Cinar E. (2022). A Sensor Fusion Method Using Transfer Learning Models for Equipment Condition Monitoring. Sensors.

